# In vivo validation of anti-malarial activity of crude extracts of *Terminalia macroptera*, a Malian medicinal plant

**DOI:** 10.1186/s12936-018-2223-7

**Published:** 2018-02-05

**Authors:** Mahamane Haidara, Mohamed Haddad, Adama Denou, Guillaume Marti, Sandra Bourgeade-Delmas, Rokia Sanogo, Geneviève Bourdy, Agnès Aubouy

**Affiliations:** 1 UMR 152 PHARMA-DEV, IRD, UPS, Université de Toulouse, Toulouse, France; 2 Faculté de Pharmacie, Université des Sciences des Techniques et des Technologies de Bamako (USTTB), BP 1805, Bamako, Mali; 3 Département de Médecine Traditionnelle de l’Institut National de Recherche en Santé, BP 1746, Bamako, Mali

**Keywords:** Antimalarial activity, *Terminalia macroptera*, In vitro, Mice models, *Plasmodium berghei* ANKA, *Plasmodium chabaudi*, Toxicity

## Abstract

**Background:**

*Plasmodium falciparum* malaria is still one of the most deadly pathology worldwide. Efficient treatment is jeopardized by parasite resistance to artemisinin and its derivatives, and by poor access to treatment in endemic regions. Anti-malarial traditional remedies still offer new tracks for identifying promising antiplasmodial molecules, and a way to ensure that all people have access to care. The present study aims to validate the traditional use of *Terminalia macroptera*, a Malian plant used in traditional medicine.

**Methods:**

*Terminalia macroptera* was collected in Mali. Leaves (TML) and roots ethanolic extracts (TMR) were prepared and tested at 2000 mg/kg for in vivo acute toxicity in Albino Swiss mice. Antiplasmodial activity of the extracts was assessed against a chloroquine resistant strain *P. falciparum* (FcB1) in vitro. In vivo, anti-malarial efficacy was assessed by a 4-day suppressive test at 100 mg/kg in two malaria murine models of uncomplicated malaria (*Plasmodium chabaudi chabaudi* infection) and cerebral malaria (*Plasmodium berghei* strain ANKA infection). Constituents of TMR were characterized by ultra-high-performance liquid chromatography coupled to high resolution mass spectrometry. Top ranked compounds were putatively identified using plant databases and in silico fragmentation pattern.

**Results:**

Lethal dose of TML and TMR were greater than 2000 mg/kg in Albino Swiss mice. According to the OECD’s Globally Harmonized System of Classification, both extracts are non-toxic orally. Antiplasmodial activity of *T. macroptera* extracts was confirmed in vitro against *P. falciparum* FcB1 strain with IC50 values of 1.2 and 1.6 µg/mL for TML and TMR, respectively. In vivo, oral administration of TML and TMR induced significant reduction of parasitaemia (37.2 and 46.4% respectively) in *P. chabaudi chabaudi* infected mice at the 7th day of infection compared to untreated mice. In the cerebral malaria experimental model, mice treated with TMR and TML presented respectively 50 and 66.7% survival rates at day 9 post-infection when all untreated mice died. Eleven major compounds were found in TMR. Among them, several molecules already known could be responsible for the antiplasmodial activity of the roots extract of *T. macroptera*.

**Conclusions:**

This study confirms both safety and anti-malarial activity of *T. macroptera*, thus validating its traditional use.

## Background

Malaria is still one of the most deadly pathology worldwide with 429,000 deaths reported in 2015 [1]. Africa is by far the most affected region, accounting for 92% of malaria deaths. Despite a recent decrease in malaria mortality due to extensive malaria control through insecticide-impregnated bed nets and increased use of artemisinin derivatives, the state of artemisinin resistance is highly worrying [2]. Artemisinin-based combination therapy constitutes the first-line treatment for malaria in the large majority of countries in which the disease is endemic, and intravenous artesunate the most efficacious treatment for severe malaria. However, *Plasmodium falciparum* has become resistant to almost all available anti-malarial drugs in Southeast Asia [3], and an isolate originating from Equatorial Guinea has been recently found resistant to artemisinin [4]. New effective molecules are thus urgently needed to combat this pathology.

Plants constitute a huge reservoir for bioactive molecules, as evidenced by the Cinchona tree and *Artemisia annua* that contain quinine and artemisinin, respectively. Africa offers an extremely rich flora and proposes numerous medicinal plants [5–7]. *Terminalia macroptera* is a Combretaceae (syn. *Terminalia chevalieri*, *Myrobalanus macroptera*) widespread in West Africa from Senegal to Cameroon [8]. In Mali, *T. macroptera* is one of the most cited plant used in traditional medicine to treat a large variety of diseases including malaria [9]. In addition, traditional remedies allow an affordable access to anti-malarial drugs for the most deprived [10–13]. First studies on *T. macroptera* have been performed in Guinea-Bissau, where extracts of roots and leaves demonstrated antibacterial activities in vitro [14–16]. Its traditional anti-malarial use was first reported from Burkina Faso; in vitro antiplasmodial activity of the total extract of *T. macroptera* was confirmed (IC_50_ = 1 µg/mL for the aqueous extract) [17]. Prepared from the plant collected in Guinea, the ethanolic root bark extract displayed a moderate antiplasmodial activity (IC_50_ = 6.8 µg/mL) [18]. However, in vivo anti-malarial activity of *T. macroptera* has not been assessed yet. Hence, this study aims to validate the traditional use of *T. macroptera* roots and leaves against malaria, a potentially fatal disease, through a dual in vivo experimental approach aiming to mimic uncomplicated malaria and cerebral malaria, the most severe form of the disease. Thus, *Plasmodium chabaudi chabaudi* and *Plasmodium berghei* strain ANKA infection in Albino Swiss mice, the respective experimental models for uncomplicated and cerebral malaria, were used to evaluate anti-malarial activity of root and leave extracts of *T. macroptera*. Finally, the nature of the major chemical compounds present in the plant was investigated.

## Methods

### Plant collection

The leaves and roots of *T. macroptera* were collected in August 2015 from Siby village in the Koulikoro region of Mali. Authorization for collection of plant materials at the Traditional Medicinal Department of the National Institute of Public Health (DMT/NIRPH) was obtained prior to collection. After collection, botanical identification was confirmed at the national herbarium of DMT/NIRPH in Bamako, Mali. A specimen of the plant with voucher number (3752/DMT) was deposited in the herbarium of DMT/NIRPH.

### Preparation of plant extracts

*Terminalia macroptera* leaves and roots were dried under shade at room temperature for 2 weeks and ground to a powder before extraction. A total of 250 g dried samples were macerated in 1 L of 90% ethanol for 24 h. The mixtures were filtered using Whatman filters No 1. The unfiltered residues were macerated in 90% ethanol for 24 h and filtered again as before. This operation was repeated three times. Filtrates were combined and evaporated in vacuo to dryness (Büchi^®^ rotary evaporator Model R-200). Crude extracts of *T. macroptera* leaves (TML) and roots (TMR) were stored in the refrigerator at 4–8 °C until use. Each extract was dissolved in methanol to provide a stock solution (10 mg/mL) kept at 4–8 °C. Before in vitro and in vivo experiments, stock solution was dissolved in distilled water to provide working concentrations.

### In vitro antiplasmodial activity

The in vitro anti-malarial activity of *T. macroptera* extracts was investigated using the SYBR Green I-based fluorescence assay [19]. The asexual intra-erythrocytic stage of *P. falciparum* laboratory strain FcB1 (chloroquine-resistant strain) was maintained in RPMI 1640 medium containing l-glutamine 200 µM, Hepes 25 mM and 5% human serum (Etablissement français du Sang—EFS, Toulouse, France) using the culture technique described by Trager and Jensen [20]. For anti-malarial drug assays, stock solutions of plant extracts were diluted serially in RPMI 1640 culture medium to test final concentrations between 1 and 100 µg/mL in triplicates in a 96-well plate. Final concentration of methanol was 0.5%. A suspension of sorbitol-synchronized, infected red blood cells (iRBCs) was adjusted to 1% parasitaemia and 2% haematocrit in complete medium and added to the wells. Negative controls were prepared with a suspension of iRBCs and 0.5% methanol. Chloroquine was used as positive control. Test plates were incubated at 37 °C for 48 h. Afterwards, 100 μL SYBR Green I fluorescent lysis buffer were added to each well and incubated in a dark place at room temperature for 2 h. Fluorescence data were acquired using a fluorescence plate reader (BMG Fluostar Galaxy Labtech) with excitation and emission wavelengths at 485 and 518 nm, respectively. The fluorescence values (after subtraction of the background fluorescence of the non-parasitized RBCs) were plotted against the log of the drug concentration, and analysed by non-linear regression (sigmoidal dose response/variable slope equation) to yield the IC50 (50% inhibitory concentration) that served as a measure of the anti-malarial activity (IVART tool, worldwide anti-malarial resistance network).

### Acute oral toxicity measurement

Oral toxicity tests were performed in the DMT/NIRPH animal house in Bamako, Mali. Female healthy Albino Swiss mice aged 6–8 weeks and weighing 26–33 g were maintained in standard and constant laboratory conditions (23–25 °C and light/dark cycles i.e. 12/12 h) with free access to food and tap water. Mice were randomly divided into three groups of three mice and treated by oral route with a single dose of TML, TMR, or water. For TML and TMR, the dose administered was 2000 mg/kg, while 20 mL/kg was used for water treatment. Oral gavage was chosen as mode of administration to mimic the traditional route of administration as described previously [21]. Oral gavage was achieved as per guidelines of the Organization for Economic Cooperation and Development (OECD) [22]. Animals were observed for the first 4 h after treatment to record immediate deaths and once daily for 14 days to record any manifestation of toxicity.

### Murine malaria models and in vivo anti-malarial tests

In vivo anti-malarial tests were realized in the animal house of PHARMADEV research unit in Toulouse, France. Mice used for these experiments were similar to the mice used for toxicity tests for age, weight and living conditions. *Plasmodium c. chabaudi* and *P. berghei* infections in female Albino Swiss mice were used as experimental models for uncomplicated and cerebral malaria, respectively [23, 24]. Swiss Albino mice are naturally resistant to *P. chabaudi* infection. The model is characterized by a peak of parasitaemia around 7 days post-infection followed by a spontaneous cure*. Plasmodium berghe*i ANKA infection leads to death in untreated mice after 7–10 days with a parasitaemia reaching 10–20% at most. In vivo antiplasmodial activity of TML and TMR was evaluated according to the 4-day suppressive standard test described by Knight and Peters [25]. Treatment was administered by oral gavage to mimic the traditional route of administration. For each model of infection, twenty-four mice were randomly divided into four treatment groups of six mice each: TMR, TML, chloroquine (positive control) and distilled water (negative control). On the 1st day (D0), mice were inoculated intraperitoneally with 0.2 mL of infected blood containing about 1 × 10^6^ parasitized erythrocytes. Two hours after infection, the two tested groups of each infection model were treated orally with 100 mg/kg of TML or TMR. Positive and negative control mice received 5 mg/kg of chloroquine and 25 mL/kg of distilled water, respectively. All mice groups were treated similarly for 4 consecutive days (D0–D3) between 9 a.m. and 10 a.m. Weight, parasitaemia and survival were followed daily. To evaluate the ability of *T. macroptera* extracts to prevent weight loss due to infection, weight differences between days post-infection and D0 were calculated. To measure parasitaemia, thin blood smears were made daily from tail blood from the 5th day (D4), until the 15th day (D14). Blood smears were fixed with methanol and stained with fast acting variation of May-Grünwald Giemsa staining (RAL 555 kit, RAL diagnostics). Parasitaemia was determined by light microscopy using a 100× objective lens as follows: % Parasitaemia = 100 × (Number of parasitized RBC/Total number of RBC counted). Average percentage chemosuppression was calculated at D7 for both infection models as [(A − B)/A] × 100 where A is the average percentage parasitaemia in the negative control group and B is the average percentage parasitaemia in the test group. Survival was monitored twice daily. The percentage survival was determined over a period of 14 days (D0–D13) and compared between groups.

### Metabolites profiling by UHPLC-HRMS

Metabolite profiles of TMR ethanol extract (1 mg/mL) were acquired using a UHPLC-DAD-CAD-LTQ Orbitrap XL instrument (Thermo Fisher Scientific, UK) equipped with an electrospray ionization source (ESI). The UHPLC system consisted of an Ultimate 3000 UHPLC (Thermo Fisher Scientific, UK) equipped with a Acquity BEH C_18_ column (100 × 2.1 mm i.d., 1.7 µm, Waters, USA). The mobile phase was composed of solvent A (0.1% formic acid–water) and solvent B (0.1% formic acid-acetonitrile) with a gradient elution (0–0.5 min, 95% A; 0.5–12 min, 95–5% A; 12–15 min, 5% A; 15–15.5 min, 5–95% A; 15.5–19 min, 95% A). The flow rate of the mobile phase was 0.45 mL/min. The injection volume was 4 µL and the column temperature was maintained at 40 °C. Electrospray ionization was applied in negative ion (NI) and positive ion (PI) mode under the following conditions: capillary voltage at 3.0 and 4.2 kV for NI and PI respectively, and capillary temperature at 300 °C. The UV detection was performed by a diode array detector (DAD) from 210 to 400 nm. Full mass spectra were recorded between 100 and 1500 Da. Collision Induced Dissociation mass spectra were obtained using the following parameters: 35% normalized collision energy, isolation width 2 Da, activation Q 0.250. External mass calibration was accomplished before starting the experiment. Hyphenation with Charged Aerosol Detector (CAD), Thermo Fisher Scientific, UK) was performed after DAD using a split 1:1 between CAD and Orbitrap.

Data processing and statistical analysis were performed as previously described [26]. Briefly, the UHPLC-HRMS raw data were processed with MS-Dial v.2.56 for mass signal extraction and peaks alignment [27]. Molecular formula prediction and compound annotation of significant features (*m/z*, RT pairs) were calculated with MS-FINDER 2.10 [28]. Only natural product databases focused on plants were selected [i.e. Universal Natural Products Database (UNPD), KNApSAc, PlantCyc, Dictionary of Natural Products (DNP, CRC press, v26:1) and CheBI]. Database interrogation was performed following a three-step process. Already known compounds belonging to the plant species were first analysed, then those belonging to the botanical family, and finally, molecules present in all plant databases were interrogated. Results were presented as a list of compounds sorted according to a score value for each match. This value encompassed uncertainty on accurate mass, the isotopic pattern score and the experimental MS/MS fragmentation mirrored to in silico fragmentation of the candidate structure.

### Statistical analysis

Results were expressed as mean ± SEM (standard error of mean) and analysed using Graph Pad Prism software version 6. Comparisons were performed by a one-way analysis of variance (ANOVA) followed by the means multiple comparison method of Bonferroni–Dunnett. Differences were considered significant if P < 0.05.

### Animals and ethics statement

Animal welfare requirements were strictly considered during the experiments as required by the National Institute for Research in Public Health (INRSP) ethics committee in Bamako, Mali, and the Midi-Pyrénées ethic committee for animal experimentation in Toulouse, France. Both studies (oral toxicity tests and anti-malarial in vivo evaluation) were authorized with permit numbers 24/2016/CE-INRSP and APAFIS#5921-2016070118008477 v3 for Bamako and Toulouse, respectively.

## Results

### Acute oral toxicity assessment and determination of the test dose

Due to the absence of previous in vivo studies on *T. macroptera* crude extracts, acute oral toxicity was first evaluated. Oral administration of TML and TMR at a dose of 2000 mg/kg did not cause mortality or major behavioural changes among experimental animals. This indicates that LD_50_ of TML and TMR are greater than 2000 mg/kg in Albino Swiss mice. Therefore, according to the OECD’s Globally Harmonized System of Classification [22], the fractions can be classified as category 5 and considered non-toxic orally. To work under the most favourable experimental conditions, *T. macroptera* extracts were used in the following experiments at 100 mg/kg for antiplasmodial activity, a dose 20 times lower.

### Effect of *T. macroptera* on weight after *Plasmodium* infection in mice

To evaluate the effect of *T. macroptera* extracts on weight following infection, analysis was first focused on weight differences between the initial weight (D0) and D4 according to the Peters’ test. Second, weight differences between D0 and D7 (peak of parasitaemia in the *P. chabaudi* model) and D0-D9 (peak of death in the *P. berghei* model) were studied. In the *P. chabaudi* model, TML-treated mice lost more weight than those treated with vehicle at D4. However, weight loss was similar between mice treated with CQ and those that received extracts. Surprisingly, the highest weight loss was observed in CQ-treated mice at D7 in this model. Comparatively, no weight loss was noticed for TML- and TMR-treated mice at this time. In the *P. berghei* infection model, TML and TMR treatments had no significant effect on weight at D4 (Table 1). At D9 when all mice that received vehicle have died, the CQ-treated mice lost less weight compared to TML- and TMR-treated mice, as shown on Table 1.

**Table 1:** Differences of body weight of *Plasmodium*-infected Swiss mice before (D0) and after infection and administration of the extracts of *T. macroptera* roots (TMR) and leaves (TML) at 4, 7 and 9 days post-infection (D4, D7, D9)

Infection group	Treatment group	n	D4–D0 ± SEM	n	D7–D0 ± SEM
*P. chabaudi chabaudi*	Vehicle	6	0.54 ± 0.22	6	0.46 ± 0.51
	CQ	6	− 0.23 ± 0.43	6	− 1.03 ± 0.48*
	TML	6	− 0.57 ± 0.43*	6	0.17 ± 0.43
	TMR	6	0.22 ± 0.19	6	0.74 ± 0.36^§^

Infection group	Treatment group	n	D4–D0	n	D9–D0
*P. berghei* ANKA	Vehicle	6	0.67 ± 0.33	0	–
	CQ	6	− 0.01 ± 0.40	5	− 0.31 ± 0.89
	TML	6	0.12 ± 0.42	4	− 2.16 ± 1.17
	TMR	6	0.11 ± 0.44	3	− 4.4 ± 1.31^§^

Chloroquine (CQ) was used as positive control

*SEM* standard error of mean

* Comparison of mice treated with CQ or extracts to those that received vehicle. * P < 0.05

^§^Comparison of mice treated with extracts to those that received CQ. ^§^P < 0.05

### In vitro and in vivo anti-parasite activity

**Fig. 1:**
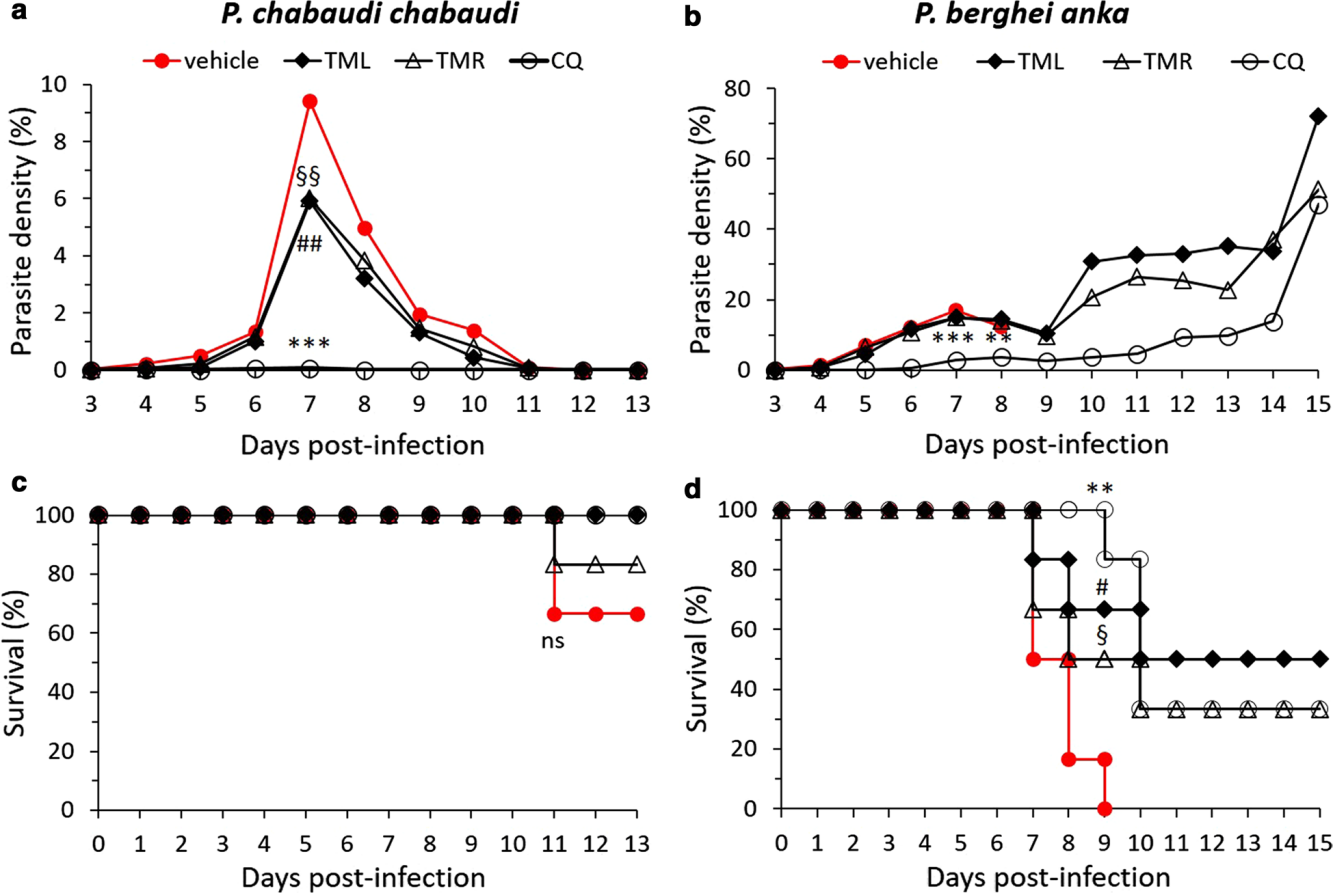
Treatment with *Terminalia macroptera* improves the outcome of *Plasmodium berghei* ANKA and *P. chabaudi chabaudi* infections. Swiss mice received vehicle (water), or extracts of leaves of *Terminalia macroptera* (TML, 100 mg/kg), or extracts of roots of *Terminalia macroptera* (TMR, 100 mg/kg), or chloroquine (CQ, 25 mg/kg) 2 h following intraperitoneal infection (10^6^ *Plasmodium*/mice) from day 0 to day 4. **a**, **b** Parasite densities were measured daily. Parasite densities were compared according to treatment received, **P < 0.005 or ***P < 0.0005 compared CQ treatment to vehicle, ^##^P < 0.005 compared TMF to vehicle, ^§§^P < 0.005 compared TMR to vehicle. **c**, **d** survival were measured daily. Survival were compared according to treatment received at D9 for *P. berghei* and at D11 for *P. c. chabaudi*, **P < 0.005 compared CQ treatment to vehicle, ^#^P < 0.05 compared TMF to vehicle, ^§^P < 0.05 compared TMR to vehicle. *ns* not significant

Antiparasite activity of *T. macroptera* crude extracts was measured both in vitro on *P. falciparum* infected red blood cells and in murine experimental models. TML and TMR showed in vitro antiplasmodial activity against FcB1 strain of *P. falciparum*, with IC_50_ values of 1.2 and 1.6 µg/mL, respectively (Table 2). The IC_50_ was 60 ng/mL for CQ. In mice models, TML and TMR were more active to lower *P. chabaudi* than *P. berghei* parasitaemia. Suppression percentages of *P. chabaudi* parasitaemia were 37.2 and 46.4%, respectively, at D7 for TML and TMR (Table 2, Fig. 1a). In addition, parasitemia was significantly lower in TML- or TMR-treated mice compared to vehicle-treated mice (Fig. 1a). In the *P. berghei* model, TML and TMR were weakly active against parasites, as parasitaemia were similar between vehicle-treated mice and TML- or TMR-treated mice (Table 2, Fig. 1b).

**Table 2:** In vivo and in vitro anti-parasite activity of the extracts of *T. macroptera* roots (TMR) and leaves (TML)

	In vitro IC_50_ (µg/mL)	% parasite suppression at D7 (± SD)	
	*P. falciparum* FcB1	*P. berghei* ANKA model	*P. chabaudi chabaudi* model
CQ	0.06	83.6 ± 6.2	99.3 ± 0.31
TML	1.2	12.2 ± 2.1	37.2 ± 5.1
TMR	1.6	13.4 ± 5.2	46.4 ± 6.8

Chloroquine (CQ) was used as positive control

*SD* standard deviation

### Effect of *T. macroptera* treatments on survival following *Plasmodium* infection in mice

In the *P. chabaudi* infection model, three mice died after the 11th day of infection including one TMR-treated mouse and two vehicle-treated mice (Fig. 1c). The resulting survival rates between groups were similar. In the *P. berghei* model, comparisons of survival rates between groups at D9 post-infection show a critical effect of TML and TMR treatments on survival (Fig. 1d). At D9, all mice that received vehicle died whereas 3 of the 6 mice (50%) treated with TMR, and 4 of the 6 mice (66.7%) treated with TML, survived (Fig. 1d). In the CQ treatment group, 5 of the 6 (83.3%) infected mice survived at D9. At D15, survival rates of TML- or TMR-treated mice were similar to those of the CQ-treated mice (3/6 and 2/6 for TML and TMR versus 2/6 for CQ, see Fig. 1d).

### Metabolite profiling of *T. macroptera* roots

For qualitative analysis by LC-CAD-MS, analysis was focused on root extracts, as root constitute the part of *T. macroptera* specifically used by traditional healers to treat malaria [21].

**Fig. 2:**
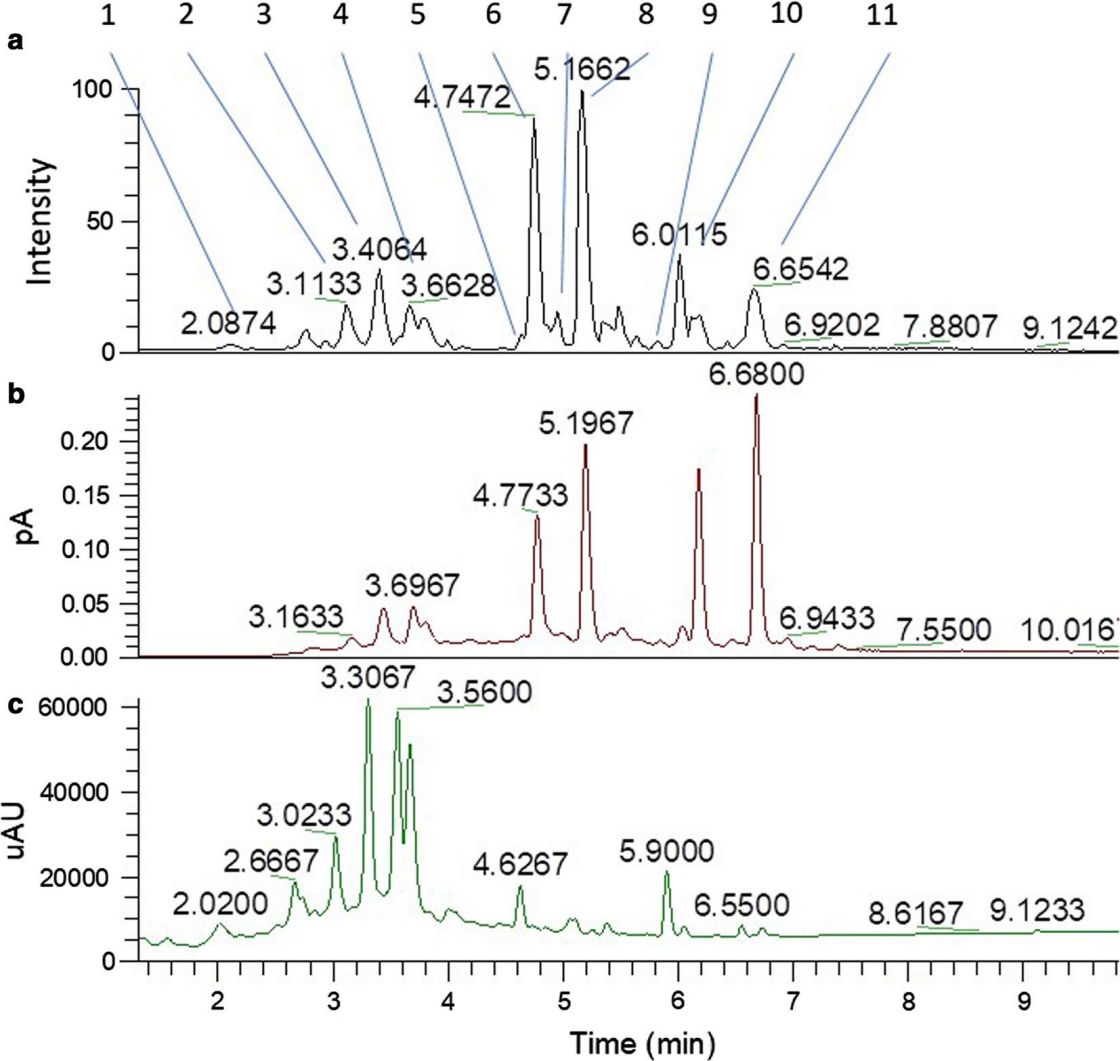
Multiplexed UHPLC chromatogram of TMR extract. **a** FTMS in ESI negative mode. **b** CAD detector. **c** PDA chromatogram (200–400 nm). Peaks numbering is according to Table 3

Metabolite profiling of *T. macroptera* roots was acquired in positive and negative ionization mode. Qualitative analysis by LC-CAD-MS of *T. macroptera* roots allowed to identify putatively 11 major peaks through HRMS and MS/MS fragmentation patterns using MS-finder and DNP database (Fig. 2, Table 3). The MS-Finder dereplication method allowed to annotate these major peaks, mostly found in the Combretaceae family (Table 3). Among the top eleven annotated compounds, 5 are tannins (flavogallonic acid, Terminalin, ellagic acid, ellagic acid; 2,8-di-me ether, 3-*O*-β-d-xylopyranoside, ellagic acid; 2,3,7-tri-me ether), 4 triterpenoids (oleaterminaloic acid C, bellericagenin B, sericic acid, oleaterminaloic acid B), 1 lignan (3,4,5-tri-*O*-caffeoylquinic acid) and 1 unknown compound.

**Table 3:** Putative identified features (m/z × RT pairs) using HRMS and MS/MS fragmentation patterns using MS-finder and DNP database

Peak no	RT (min)	m/z	MF	Δ Da	Putative ID	Found in	Chemical class
1	2.13	468.997 [M−H]^−^	C_21_H_10_O_13_	0.007	Flavogallonic acid	*T. catappa* (Combretaceae)	Tannins
2	3.11	541.0167	C_30_H_18_O_12_	0.006	Unknown		
**3**	**3.4**	**603.0075 [M+H]** ^**+**^	**C**_**28**_ **H**_**10**_ **O**_**16**_	**0.005**	**Terminalin**	***T. oblongata*** **(Combretaceae)**	**Tannins**
4	3.66	303.0150 [M+H]^+^	C_14_H_6_O_8_	0.001	Ellagic acid	Many plants	Tannins
5	4.55	461.0644 [M−H]^−^	C_21_H_18_O_12_	0.008	Ellagic acid; 2,8-di-me ether, 3-*O*-β-d-xylopyranoside	*T. superba* (Combretaceae)	Tannins
**6**	**4.75**	**505.3547 [M+H]** ^**+**^	**C**_**30**_ **H**_**48**_ **O**_**6**_	**0.003**	**Oleaterminaloic acid C**	***T. arjuna*** **(Combretaceae)**	**Triterpenoids**
7	4.9	519.3235 [M−H]^−^	C_30_H_48_O_7_	0.009	Bellericagenin B	*T. bellerica*; *T. arjuna* (Combretaceae)	Triterpenoids
**8**	**5.16**	**695.1531 [M−H]** ^**−**^	**C**_**34**_ **H**_**30**_ **O**_**15**_	**0.009**	**3,4,5-Tri-** ***O*** **-caffeoylquinic acid**	**Many plants**	**Lignans**
9	5.89	501.3218 [M−H]^−^	C_30_H_48_O_6_	0.0003	Sericic acid	Many *Terminalia* sp.	Triterpenoids
**10**	**6.01**	**343.0411 [M−H]** ^**−**^	**C**_**17**_ **H**_**12**_ **O**_**8**_	**0.005**	**Ellagic acid; 2,3,7-tri-me ether**	**Many plants**	**Tannins**
**11**	**6.65**	**487.3343 [M+H]** ^**+**^	**C**_**30**_ **H**_**46**_ **O**_**5**_	**0.0003**	**Oleaterminaloic acid B**	***T. arjuna*** **(Combretaceae)**	**Triterpenoids**

Major peaks detected by CAD are in bold

## Discussion

Access to treatment remains a major health problem in sub-Saharan Africa. To face this critical public health problem and improve the management of malaria cases, home-based management is the most implemented measure [29, 30], but the use of traditional medicine in a safe, cost-efficient and effective manner also constitutes a way to ensure that all people have access to care. The World Health Organization (WHO) traditional medicine strategy 2014–2023 reminds that traditional medicine is found worldwide, and that its use is constantly increasing [31]. The use of traditional medicine is particularly rooted in African culture and history, although scientific validation of traditional remedies is still scarce [32]. Among the strategic objectives of the WHO, the strengthening of safety and effectiveness of traditional medicine constitutes one of the main validation before integrating traditional medicine in health systems. In Mali, *T. macroptera* is used traditionally in the treatment many diseases including malaria, inflammatory diseases and liver diseases [21, 33, 34]. However, in vivo validation of safety and efficiency has never been achieved. The present study aimed to evaluate toxicity and anti-malarial efficacy of crude extract of this plant to validate its traditional use.

The results of the acute toxicity tests indicate that administration of up to 2 g/kg of ethanolic extracts of TML and TMR is safe in Albino Swiss mice. Thus, the dose tested in this study (100 mg/kg) was safe, and it can be assumed that the anti-malarial activities observed are not due to toxicity.

In vivo tests were achieved in two different experimental models for malaria based on *P. chabaudi* and *P. berghei* infections in Swiss mice. Both models reflect two different clinical types of malaria observed in humans: uncomplicated and cerebral malaria, respectively [23, 24]. In the 4-day suppressive test performed in the *P. chabaudi* model, weight loss was higher in TML-treated mice compared to those that received CQ, suggesting a depressing effect of this crude extract on food intake or appetite. At D7, when *P. chabaudi* parasitaemia was the highest, CQ-treated mice controlled their parasitaemia and presented a significant weight loss compared to the other mice groups, suggesting that weight loss at D7 is not a key criterion in this model. In the most severe malaria model with *P. berghei*, administration of TML or TMR to mice induced similar weight changes compared to CQ or vehicle at D4, strengthening the safety of TML and TMR at the tested doses. Weight loss differences were observed between treatments at D9 when all untreated mice have died. CQ induced a lower weight loss compared to plant extracts. This result suggests that *T. macroptera* extracts might not reduce the deleterious effect of the parasite in this model at a critical time for survival. It would be interesting to test a longer treatment with these plant extracts in this model.

To verify the direct antiparasite activity of *T. macroptera*, extracts were initially screened for their in vitro activity against *P. falciparum* FcB1 strain. IC_50_ values were 1.2 and 1.6 µg/mL for TML and TMR, respectively. These values reflect a good antiplasmodial activity in vitro, assuming that IC50 < 10 µg/mL constitutes an accurate threshold for active plant extracts [35]. In line with the results observed, similar IC50 values were obtained for root crude extracts of *T. macroptera* in two independent studies from Burkina Faso and Guinea [17, 18]. In these studies, extracts displayed IC50 values between 1 and 6.8 μg/mL against *P. falciparum* K1 strain, a chloroquine-resistant strain as FcB1.

Antiplasmodial activity of *T. macroptera* extracts was confirmed in vivo in the *P. chabaudi* model. TML- and TMR-treated mice displayed about 40% chemosuppression at D7 when the peak of parasitaemia was reached in untreated mice. This significant result reflects an inhibitory activity on parasite replication in this model. This activity was not verified in the *P. berghei* model. However, significant increased survival of *P. berghei* infected mice treated with plant extracts and CQ was obtained in comparison with untreated mice. At D9, all untreated mice died whereas 66.7 and 50% of mice that received TML or TMR survived, respectively. This result indicates a very interesting ability to extend survival in this model characterized by a high mortality rate. However, survival to *P. berghei* infection was not related to parasite elimination in TML- and TMR-treated mice. In this model, death is due to neuroinflammation related to influx of myeloid immune cells to the brain, oxidative stress, blood brain barrier permeability and neurodegeneration [36, 37]. Previous in vitro data demonstrated that *T. macroptera* extracts display anti-oxidant properties through radical scavenging and α-glucosidase inhibition [38–40]. Therefore, further in vivo studies would be of high interest to verify that survival of *P. berghei*-infected mice after *T. macroptera* treatment relies on the anti-oxidant properties of the plant.

Qualitative analysis by LC-CAD-MS of *T. macroptera* roots allowed to identify putatively 11 major peaks (Fig. 2, Table 3). The results are confirmed by several studies that have shown the presence of gallic acid, punicalagin, terflavin A, terchebulin, ellagic acid and their methoxylated derivatives in the roots of *T. macroptera* [41, 42]. Of the 11 compounds identified, only the antiplasmodial properties of flavogallonic acid and ellagic acid have been reported in the literature. These studies have shown moderate in vitro antiplasmodial activities on *P. falciparum* K1 and 3D7 strains (IC_50_ of 8.35 and 8.89 µg/mL, respectively) of flavogallonic acid with a selectivity index greater than 168 on cells derived from the heart of newborn mice (NBMH) [43]. In addition, Verotta et al. showed that ellagic acid isolated from *Tristaniopsis callobuxus* (Myrtaceae) had significant antiplasmodial activity with an IC_50_ between 0.331 and 0.480 µM, regardless of the level of resistance of the *Plasmodium* strain used [44]. Similar results were obtained by Banzouzi et al. with isolated ellagic acid from *Alchornea cordifolia* (Euphorbiaceae) [45] and by Soh et al. with commercial ellagic acid [46]. The antiplasmodial activity of ellagic acid has also been demonstrated in vivo in mice infected with *Plasmodium vinckei petteri* using the Peters assay. Soh et al. demonstrated that ellagic acid inhibits parasitaemia when administered intraperitoneally at doses of 1, 50 and 100 mg/kg/day with more than 50% inhibition at the 1 mg/kg/day dose and 100% inhibition at the 50 and 100 mg/kg/day dose [46]. In addition, antioxidant activity of ellagic acid was recently demonstrated in a murine model of cirrhosis [47]. Such activity is of high importance in the survival of *P. berghei*-infected mice and could explain the higher survival rate obtained after *T. macroptera* treatment. Although these results brought interesting hypothesis regarding putative active compounds found in *T. macroptera*, further bioassay guided fractionation will be necessary to decipher pharmacological basis of its antiplasmodial activity including synergistic potential between tanins, lignans and terpenoids found in this plant.

## Conclusions

The West-African plant *T. macroptera* demonstrated in this study in vitro and in vivo anti-malarial activities on two murine malaria models, including the experimental cerebral malaria model. This plant constitutes a promising starting point for bioguided isolation of new anti-malarial molecules that could be useful for both uncomplicated and severe malaria treatments. Finally, this study confirms both safety and anti-malarial activity of *T. macroptera*, thus validating its traditional use in West Africa.

## Abbreviations

dH_2_O: distilled water
D: day
DMT/NIRPH: Traditional Medicinal Department/National Institute of Public Health
IC50: 50% inhibitory concentration
RBC: red blood cell
SEM: standard error of mean
TML: *T. macroptera* leaves
TMR: *T. macroptera* roots
WHO: World Health Organization
OECD: Organization for Economic Cooperation and Development
